# Telovelar Approach for the Surgical Resection of a Caudal Fossa Glioma in a Toy Poodle

**DOI:** 10.3390/ani15091240

**Published:** 2025-04-28

**Authors:** Victoria Kymm, Youngjin Jeon, Il-Hwa Hong, Yoonho Roh

**Affiliations:** 1 Institute of Animal Medicine, Department of Surgery, College of Veterinary Medicine, Gyeongsang National University (GNU), Jinju 52828, Republic of Korea; vtk@gnu.ac.kr; 2 Department of Veterinary Surgery, College of Veterinary Medicine, Chungnam National University, Daejeon 34134, Republic of Korea; orangee0115@gmail.com; 3 Institute of Animal Medicine, Department of Veterinary Pathology, College of Veterinary Medicine, Gyeongsang National University (GNU), Jinju 52828, Republic of Korea; ihhong@gnu.ac.kr

**Keywords:** caudal fossa, glioma, telovelar approach, fourth ventricle

## Abstract

A 7-year-old, 6.5 kg, neutered male toy poodle presented with tetraparesis, characterized by lower motor neuron signs in the forelimbs and upper motor neuron signs in the hindlimbs, along with seizures. Diagnostic imaging using magnetic resonance imaging (MRI) and computed tomography (CT) revealed a 1.4 cm × 1.4 cm × 2.2 cm mass in the fourth ventricle and caudal part of the brainstem. The surgical objective was to precisely remove masses compressing the cerebellum and brainstem. Using the telovelar approach, the tumor was partially excised, contrary to the goal of complete removal. Histopathological analysis confirmed the diagnosis of glioma. By the third postoperative day, the patient began to walk independently, and tetra-ataxia symptoms gradually decreased. Postoperative imaging confirmed the successful debulking of the tumor. By postoperative day 15, the patient showed normal gait, and adjuvant radiation therapy (RT) was initiated 2 weeks later. Unfortunately, the patient died 91 days after surgery, though the precise cause of death remains undetermined.

## Simple Summary

This case report describes a rare condition in a dog diagnosed with a caudal fossa glioma, which was treated using the telovelar approach. This surgical method has not been previously reported for glioma of the caudal fossa. The application of this surgical technique provides valuable information regarding patient prognosis in veterinary neurosurgery.

## 1. Introduction

Intracranial gliomas in dogs are primary brain tumors originating from glial cells and can occur anywhere within the brain parenchyma, though they are particularly prevalent in the hemispheres [1,2]. In addition to the hemispheres, gliomas may also arise in the diencephalon and infratentorial region [2,3]. Gliomas are the second most common brain tumor in dogs, with an incidence rate of 36% [4,5,6]. These tumors exhibit histopathological characteristics similar to those of human gliomas and can be classified into several subtypes, including oligodendroglioma, astrocytoma, and undefined glioma (previously oligoastrocytoma or mixed glioma) [1,6].

In humans, gliomas commonly present with cognitive impairment (57%), various types of seizures (52.8%), and aphasia (25.4%) [7]. These symptoms, particularly cognitive impairment and aphasia, can be difficult to assess for dog owners. Dogs with gliomas typically present with more observable symptoms, such as proprioceptive deficits (74%), seizures (61%, with 35% experiencing isolated seizures and 26% having cluster seizures), and abnormal gait (59%), including ataxia or paresis [1,3,6,8]. Given that other brain tumors can present with similar symptoms, diagnosing gliomas based on clinical signs alone is challenging [5].

In veterinary medicine, treatment protocols for intraventricular tumors are not well-established, whereas in human medicine, surgical removal is considered the most effective treatment option [8,9]. Fourth ventricle tumors are typically resected via the transvermian or telovelar approach [9,10,11,12]. The transvermian approach, which requires splitting the inferior vermis, has been associated with complications such as cerebellar mutism and dysfunction in humans [11,12]. Conversely, the telovelar approach could preserve vermian integrity by accessing the fourth ventricle between the medullary velum and cerebellar tonsils, offering wider exposure and reduced risk of neurological deficits [10,11,12,13,14,15,16]. Other approaches, such as the superior transvelar and infradentate techniques, have been described. The superior transvelar allows access to the upper fourth ventricle but requires tentorial incision and a deep trajectory [17]. The infradentate is minimally invasive and preserves critical structures but demands specialized equipment and advanced skill [16,18]. Currently, the telovelar approach remains the only technique reported in veterinary medicine [9,19].

To the best of our knowledge, there have been cases of complete removal of choroid plexus tumors and meningiomas from the fourth ventricle using the telovelar approach [9,19]. However, there are no reported instances of using this approach to remove glioma, followed by complete radiotherapy. This is the first report on the application of the telovelar approach for the surgical treatment of a caudal fossa glioma involving the fourth ventricle and its impact on patient outcomes following both surgery and complete radiation therapy (RT).

## 2. Case Presentation

### 2.1. History and Clinical Examination

A 7-year-old castrated male poodle weighing 6.5 kg and living indoors was referred for surgical resection of a brain tumor identified at the referring veterinary clinic. The patient experienced tonic-clonic seizures one week before being referred to the animal medical center. Before the onset of seizures, preictal symptoms included ataxia, stumbling, and knuckling, followed by limb rigidity. A few hours later, a generalized tonic-clonic seizure lasting 10–30 s occurred with loss of consciousness. During the postictal phase, the patient presented with depression and was unable to ambulate independently. Physical examination revealed a normal body temperature, respiratory rate, and blood pressure. However, the dog exhibited bradycardia, with a heart rate of 66 bpm. The patient showed tetraparesis, characterized by lower motor neuron (LMN) signs in the forelimbs and upper motor neuron (UMN) signs in the hindlimbs. The patient also exhibited lethargy and anorexia.

The patient underwent comprehensive testing, including a chemistry panel, complete blood count (CBC), and venous blood gas analysis, with blood samples collected from a vein. Additional tests included ammonia, symmetric dimethylarginine (SDMA), C-reactive protein (CRP), and coagulation profiles. Results showed an elevated alanine transaminase (ALT) level at 222 U/L (normal range 10–125 U/L), hypoamylasemia with a reading of 301 U/L (normal range 500–1500 U/L), hyperlactatemia at 3.5 mmol/L (normal range 0.5–2.5 mmol/L), and packed cell volume (PCV) was 43.5% (normal range 37.3–61.7%), with no other significant findings reported.

Following the seizures, the patient was hospitalized for four days at the referral hospital, receiving intravenous fluids at a rate of 1.25 mL/kg/h through Hartmann’s solution (Hartman Sol. (500 mL/bag), JW Pharmaceutical, Gyungi, Republic of Korea), mannitol (Dai Han D-Mannitol Injection (15%), Dai Han Pharm. Co., Seoul, Republic of Korea) at 0.25 g/kg IV CRI twice a day, and prednisolone (Samu prednisolone injection, Samu median Co., Chungcheongnam, Republic of Korea) at 0.5 kg/kg SC twice a day. Additionally, the dog was orally administered silymarin (10 mg/kg) and biphenyl dimethyl dicarboxylate (6.25 mg/dog PO; Lefortil Tab, CMG Pharm Co., Gyungi, Republic of Korea) twice daily. After stabilization, the patient was transferred to our facility for further care.

**Figure 1:**
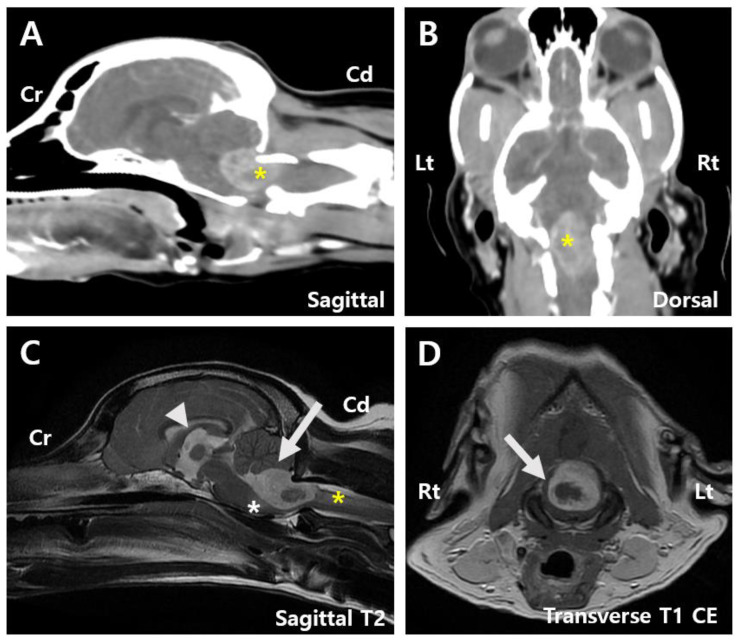
Pre-surgery images. (**A**) Sagittal computed tomography (CT) image of the patient showing a tumor measuring 1.4 cm × 2.2 cm (asterisk). (**B**) Dorsal CT image showing a tumor measuring 1.4 cm × 2.2 cm (asterisk). (**C**) Sagittal T2-weighted magnetic resonance imaging (MRI) image displaying mild dilation of the 3rd ventricle (arrowhead), compression of the cerebellum by the mass (arrow), and edematous changes extending to the adjacent brainstem (white asterisk) and C2 spinal cord (yellow asterisk). (**D**) Transverse T1-weighted contrast-enhancement magnetic resonance imaging (MRI) image suggesting a hematoma within the tumor (arrow). Cd, Caudal; CE, Contrast-enhancement; Cr, Cranial; Lt, left; Rt, right.

The referring clinic had previously performed magnetic resonance imaging (MRI) of the brain and spine, revealing a 1.4 cm × 1.4 cm × 2.2 cm mass at the level of the fourth ventricle and caudal part of the brainstem. The mass showed hyperintensity on T2-weighted (T2) images, hypointensity on T1-weighted (T1) images with internal areas of hypointensity on T2 images, and isointensity on T1 images. In addition, the mass showed peripheral contrast enhancement with an internal non-enhancing hypointensity (Figure 1D). A T2-star signal void was also observed, suggesting the presence of a hematoma.

The mass compressed the cerebellum and caused edematous changes in the adjacent brainstem and C2 spinal cord (Figure 1C). Mild dilation of the third ventricle and thinning of the arachnoid space across the cerebrum were also noted, indicating a high likelihood of an intra-axial tumor with associated swelling and compression. Computed tomography (CT) revealed heterogeneous contrast enhancement in the fourth ventricle and caudal brainstem, consistent in size with MRI findings (Figure 1A,B). Based on brain MRI and CT findings, the lesion was anatomically positioned to possibly originate from the fourth ventricle, suggesting a choroid plexus tumor or ependymoma. However, other possibilities, such as meningioma and glioma, could not be ruled out. Owing to the tumor’s location and the risk associated with overall increased intracranial pressure, cerebrospinal fluid paracentesis was not performed. No abnormalities or signs of metastasis were observed on chest radiography, CT, or cardiac ultrasonography.

### 2.2. Preoperative Management

The patient received preoperative medications including prednisolone (0.5 mg/kg PO BID; Solondo Tab, Yuhanyanghang, Seoul, Republic of Korea), gabapentin (10 mg/kg PO BID; Gabapentin Capsule 100 mg, Dong-A Pharm. Co., Seoul, Republic of Korea), and omeprazole (0.5 mg/kg PO BID; Omed Table 20 mg, SK Chemicals, Gyungi, Republic of Korea). Mannitol (0.5 g/kg IV; Dai Han D-Mannitol Injection (15%), Dai Han Pharm. Co., Seoul, Republic of Korea) was administered slowly. For pre-anesthetic preparation, the patient was administered vitamin K (3 mg/kg SC; Vitamin K1 Injection 10 mg, Dai Han Pharm. Co., Seoul, Republic of Korea) to minimize intraoperative bleeding risk, maropitant (1 mg/kg SC; Cerenia, Zoetis, Seoul, Republic of Korea) to prevent vomiting, and levetiracetam (20 mg/kg IV; Keppra Injection, UCB Pharm. Co., Seoul, Republic of Korea) as an antiepileptic drug. Additional preoperative medications included butorphanol (0.2. mg/kg, IV; Butophan Injection 1 mg/mL, Myung Moon Pharm. Co., Gyungi, Republic of Korea), midazolam (0.2 mg/kg IV; Midazolam Injection 3 mg/3 mL, Bukwang Pharm. Co., Republic of Korea), and cefazolin (25 mg/kg IV; Cefazole injection 1 g, Korea Korus, Chungbuk, Republic of Korea). Induction was performed using propofol (4 mg/kg IV; Freefol-MCT Injection, Daewon Pharm. Co., Seoul, Republic of Korea). Preparation for surgery was completed by shaving the patient’s head from the top down to the C4 region and then thoroughly disinfecting the area. For intraoperative pain management, a combination of dexmedetomidine (0.0005 mg/kg/h IV; Medex Injection, Il Sung IS, Gyungi, Republic of Korea), lidocaine (3 mg/kg/h IV; Dai Han Lidocaine HCl Hydrate Injection 2%, Dai Han Pharm. Co., Seoul, Republic of Korea), and ketamine (0.6 mg/kg/h IV; Ketamine KCl Injection, Huons, Gyungi, Republic of Korea), referred to as DLK, was administered as a continuous rate infusion (CRI). Prior to this CRI, loading doses of dexmedetomidine (0.0001 mg/kg IV), lidocaine (1 mg/kg IV), and ketamine (0.5 mg/kg IV) were given to the patient.

### 2.3. Surgical Management

**Figure 2:**
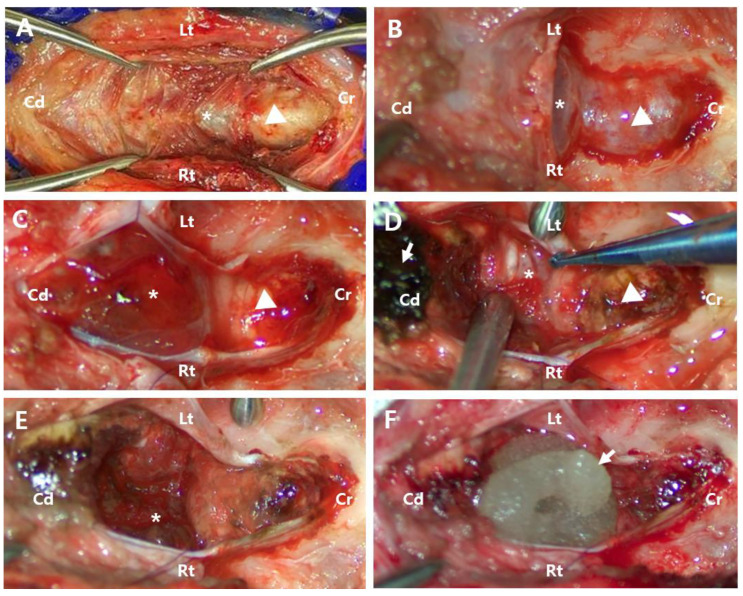
Perioperative images. (**A**) After separating the skin and muscle, the occipital bone (arrowhead) and C1 vertebra (asterisk) were exposed. (**B**) Following suboccipital craniotomy and partial dorsal laminectomy of C1, the tumor (asterisk) and cerebellar vermis (arrowhead) were covered by the dura mater. (**C**) After incising the dura mater, the dusky tan-to-pink color of abnormal tissue (asterisk) and the cerebellar vermis with a clear pink color (arrowhead) were exposed. (**D**) The tumor was infiltrative (asterisk), making it difficult to distinguish it from normal tissue. The cerebellar vermis was discolored (arrowhead) due to the application of absorbable oxidized regenerated cellulose (arrow). Absorbable oxidized regenerated cellulose with absorbed blood (arrow). (**E**) After partial tumor removal, remnants of the tumor (asterisk) were still observed. (**F**) The application of flowable thrombin material (arrow) for hemostasis after tumor resection. Cd, Caudal; Cr, Cranial; Lt, left; Rt, right.

A telovelar approach was selected to access the tumor located in the caudal part of the fourth ventricle and brainstem. The patient was positioned in ventral recumbency with the head ventroflexed at 90° to the spine. A reinforced endotracheal tube was used to prevent airway compression caused by head flexion. A midline skin incision was made from the occipital inion to the spinous process of C2 (Figure 2A). Using Kerrison rongeurs and a nitrogen-powered high-speed drill (Surgairtome Two Hall^®^, ConMed, Utica, NY, USA), a suboccipital craniotomy and partial C1 dorsal laminectomy were performed, exposing a wide dura mater (Figure 2B). A neurosurgical microscope (Leica M530 OHX, Leica Microsystems Inc., Deerfield, IL, USA) was used to enhance visualization. A Y-shaped durotomy was performed with a Von Graefe knife (Von Graefe Knife, Fine Science Tool, Foster City, CA, USA), and the dura mater was laterally retracted using polydioxanone sutures (PDS II 6/0, Ethicon, Neuchâtel, Switzerland).

Following incision, the cerebellar vermis and significant tumor protrusions were exposed, making cerebellar tonsil retraction unnecessary (Figure 2C). An incision in the tumor capsule revealed a dusky tan-to-pink mass with abnormal coloration and significant hemorrhage. The tumor had extensively invaded the parenchyma surrounding the fourth ventricle, making it difficult to distinguish between anatomical structures and precluding complete resection. Given the severity of the hemorrhage and extensive infiltration (Figure 2D), the risk of perioperative mortality was considered high, and euthanasia was recommended to the owner. However, at the owner’s request, the surgery proceeded. The tumor was meticulously dissected and excised using a micro nerve hook probe (45° Teardrop Dissector, Qswtitan, Chengdu, China), microcurette (Micro Curette, Qswtitan, Chengdu, China), and tumor forceps (Yasargil Tumor Forcep, Ruggles^®^-Redmond™, Princeton, NJ, USA). All accessible tumor tissues were removed as completely as possible, while residual tumor tissue remained caudoventral to the vermis (Figure 2E). Hemostasis was achieved intraoperatively using cold saline lavage, a microsurgical sponge (PVA spears, Koryo Eyetech, Gyungi, Republic of Korea), absorbable oxidized regenerated cellulose (Surgicel^®^, Ethicon, Neuchâtel, Switzerland), and flowable thrombin material (Floseal Hemostatic Matrix 5 mL, Baxter, Deerfield, IL, USA) (Figure 2F). Due to significant blood loss, a blood transfusion was required. Chlorpheniramine (0.2 mg/kg SC; Chlorpheniramine Maleate Injection, Huons, Gyungi, Republic of Korea) was administered beforehand to prevent potential transfusion-related reactions. Over three hours, 352 mL of DEA1-negative whole blood with a donor PCV of 40% was transfused without complications during or after the procedure. After the transfusion, the patient’s PCV was measured at 39%, showing a 4.5% decrease compared to the preoperative value. Following tumor removal, the fourth ventricle was irrigated with Hartmann’s solution to ensure no residual bleeding remained. The incised tela choroidea was left open, and the dura mater was closed using a simple interrupted suture pattern with polydioxanone sutures (PDS II 6/0, Ethicon, Neuchâtel, Switzerland). The muscle and skin layers were then securely closed.

### 2.4. Postoperative Management

On the first postoperative day, the patient’s primary symptoms, including tetraparesis and seizures, had resolved, allowing independent walking and eating. However, due to insufficient limb strength, normal ambulation was still not possible. During the postsurgical hospital stay, the patient was administered DLK CRI and butorphanol (0.2 mg/kg IV) for pain management, along with prophylactic antibiotics, cefazolin (25 mg/kg IV TID) and enrofloxacin (5 mg/kg IV SID; Baytril™ 50 Injection, Elanco, Greenfield, IN, USA). Pantoprazole (1 mg/kg IV BID; Pantoloc^®^ Injection, Takeda Pharm. Korea Co., Seoul, Republic of Korea) was administered for gastric acid suppression, and prednisolone (0.5 mg/kg IV BID) was prescribed as a corticosteroid. Maropitant (1 mg/kg IV) and levetiracetam (20 mg/kg IV TID) were also administered. However, the patient showed signs of the Cushing reflex, characterized by increased systolic arterial pressure up to 189 mmHg, decreased heart rate down to 66 bpm, and anxiety, prompting the addition of mannitol (0.25 g/kg IV) and midazolam (0.2 mg/kg IV) through CRI [20]. Clinical symptoms improved with additional medication, allowing discontinuation of mannitol and midazolam after two days. At the time of discharge, the following medications were prescribed: prednisolone (0.5 mg/kg PO BID), cephalexin (25 mg/kg PO TID; Wonkwang Cefalexin Capsule 500 mg, Won Pharm. Co., Jeolla, Republic of Korea), omeprazole (1 mg/kg PO BID), levetiracetam (20 mg/kg PO TID; Keppra Tablet 500 mg, Korea UCB Co., Seoul, Republic of Korea), enrofloxacin (10 mg/kg PO SID; Baytril Flavour, Bayer, Leverkusen, Germany), silymarin (5 mg/kg PO BID; Legalon Capsule 140, Bugwang Pharm. Co., Seoul, Republic of Korea), and biphenyl dimethyl dicarboxylate (0.5 tablet/dog PO BID). The patient exhibited mild ataxia with an unsteady gait and persistent weakness in all four limbs. However, spontaneous ambulation was observed, and the patient demonstrated responsiveness to the owner’s call by attempting to approach them. Eight days after discharge, the prednisolone dose was reduced to 0.375 mg/kg BID PO and continued for two weeks before discontinuation. Upon returning to the hospital on the 15th postoperative day for suture removal appointment, the surgical site had completely healed, and the sutures were removed. Gait examination revealed that the patient regained sufficient limb strength and was able to walk without ataxia.

### 2.5. Postoperative CT and MRI Imaging Results

**Figure 3:**
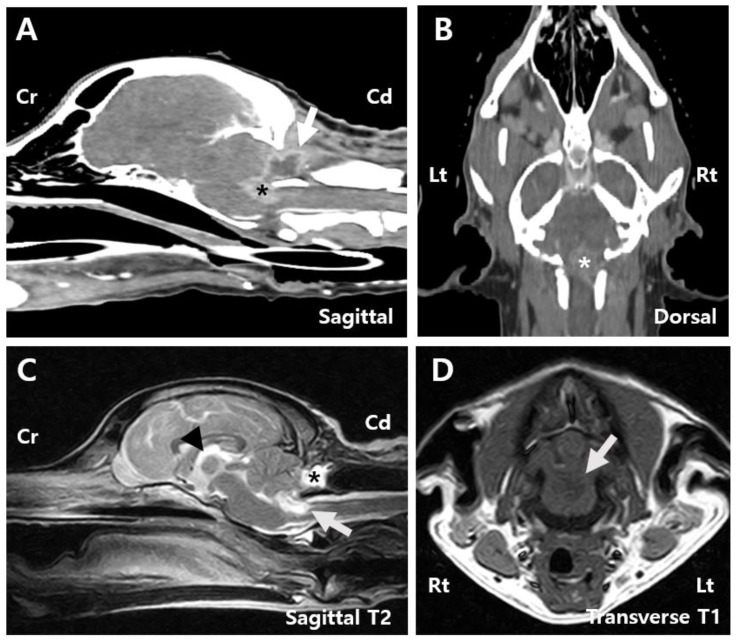
Post-surgery images. (**A**) Sagittal CT image of the patient reveals a contrast-enhancing tumor measuring 0.9 cm × 0.7 cm (arrow), along with a peripheral rim-enhancing and fluid-attenuating lesion identified within the rectus capitis dorsalis muscle (asterisk). (**B**) Dorsal CT image showing a contrast-enhancing tumor measuring 0.9 cm × 1.3 cm (asterisk). (**C**) Sagittal T2 MRI image showing peripheral T2 hyperintense lesions surrounding the mass (white arrow), a reduction in the previously dilated third ventricle (arrowhead), and a contrast-enhancing round to oval-shaped lesion measuring 1.8 cm × 0.8 cm × 1.0 cm surrounded by the overlying muscles including the rectus capitis dorsalis (asterisk). (**D**) Transverse T1 MRI image showing hypointense lesions (white arrow). Cd, Caudal; Cr, Cranial; Lt, left; Rt, right.

Postoperative CT indicated a lesion with heterogeneous contrast enhancement at the cranial aspect of the foramen magnum, potentially related to the patient’s history of a fourth ventricle tumor resection, suggesting a residual tumor lesion (Figure 3A,B). Postsurgical changes were observed at the level of the internal occipital crest and dorsal subquadrant. Peripheral rim-enhancing and fluid-attenuating lesions were identified in the rectus capitis dorsalis muscle (Figure 3A).

MRI findings showed that the mass within the fourth ventricle and the peripheral region was characterized by T2 hyperintensity, T1 hypointensity, and contrast enhancement. The mass had decreased to approximately 19% of its original size and measured 0.9 cm × 0.7 cm × 1.3 cm (Figure 3C,D). Compression of the cerebellum and medulla oblongata was also reduced. The subarachnoid space thinning that was observed preoperatively was no longer present. Additionally, a well-defined, round to oval-shaped lesion measuring 1.8 cm × 0.8 cm × 1.0 cm was identified within the rectus capitis dorsalis muscle, adjacent to the tuber and pyramid of the cerebellar vermis. The lesion exhibited T2 and FLAIR hyperintensities, T1 hypointensity, and peripheral contrast enhancement (Figure 3C). A reduction in third ventricle dilation was also noted compared to preoperative imaging (Figure 3C).

### 2.6. Postoperative Histopathology Results

**Figure 4:**
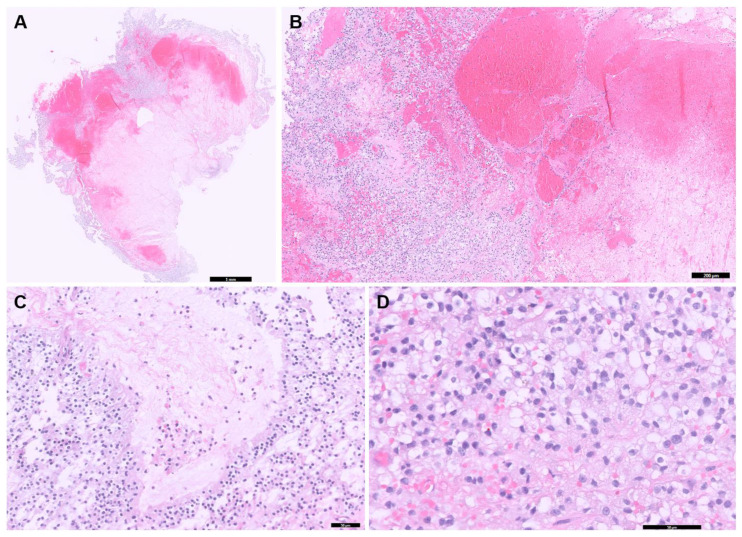
Histopathologic examination of the tumor in the brain. (**A**) Entire tissue section showing extensive areas of hemorrhage and necrosis (scale bar = 1 mm). (**B**) Extensive hemorrhage and necrosis are seen throughout the tumor (scale bar = 200 μm). (**C**) Neoplastic cells aligned perpendicularly to necrotic regions, forming a pseudo-palisading pattern (scale bar = 50 μm). (**D**) A monomorphic proliferation of round to polygonal cells exhibited variably distinct cell margins, pale, vacuolated to granular eosinophilic cytoplasm is observed (scale bar = 50 μm).

The excised tumor tissue was sent to a board-certified pathologist for histopathological analysis and additional immunohistochemistry (IHC) testing, including markers for cytokeratin AE1/AE3, vimentin, oligodendrocyte transcription factor 2 (Olig2), and glial fibrillary acidic protein (GFAP). Histopathological examination revealed that the tumor mass predominantly consisted of central hemorrhage with microvascular proliferation and necrosis surrounded by a relatively monomorphic proliferation of round to polygonal cells (Figure 4A,B). These neoplastic cells were arranged perpendicularly to necrotic regions, forming a pseudo-palisading pattern (Figure 4C). The cells exhibited variably distinct cell margins, pale, finely vacuolated to granular eosinophilic cytoplasm, and round to oval nuclei with finely stippled to dense chromatin and indistinct or occasionally prominent nucleoli (Figure 4D). Minimal anisokaryosis and low mitotic activity were also observed. A few numbers of hemosiderin-laden macrophages and polymorphonuclear cells were scattered throughout the tumor. This was likely due to a secondary inflammatory response associated with hemorrhage and necrosis. IHC results were as follows: cytokeratin AE1/AE3 showed negative immunoreactivity, vimentin demonstrated strong and diffuse cytoplasmic immunoreactivity, Olig2 showed variable nuclear immunoreactivity, and GFAP showed variable cytoplasmic immunoreactivity. Based on these findings, the tumor was classified as an undefined glioma, which is high grade due to extensive necrosis and microvascular proliferation. Additionally, the histological tumor assessment confirmed incomplete tumor excision.

### 2.7. Postoperative Radiation Therapy

To manage the residual tumor, RT was recommended, and the patient was referred to another hospital for continued treatment. RT was initiated 29 days postoperatively and continued for five weeks. The patient received 20 sessions of RT based on a hyperfractionated protocol, delivering a total dose of 50 Gy in 20 fractions with 2.5 Gy administered per session. The treatment schedule was adjusted based on the patient’s condition. During RT, ataxia was observed; however, all 20 sessions of RT were completed, and clinical symptoms improved. Post-RT CT and MRI were not performed at the owners’ discretion. The patient died 91 days after surgery. The clinical symptoms prior to death were unknown, and a necropsy was not performed; therefore, the cause of the patient’s death could not be definitively determined.

## 3. Discussion

This case study describes the surgical removal of a glioma located in the caudal fossa, involving the fourth ventricle and its surrounding tissues, using a telovelar approach. This is the first reported case of surgical excision of a caudal fossa glioma, followed by RT. Gliomas are commonly diagnosed in male dogs older than eight years [1,4]. In this case, a 7-year-old neutered male poodle exhibited symptoms of tetraparesis and seizures and was brought to our clinic.

Fourth ventricular choroid plexus tumors (CPTs) commonly appear hypointense on T1-weighted MRI and hyperintense on T2-weighted images, with strong, uniform contrast enhancement [8,21]. They most frequently occur in the fourth ventricle (49%), followed by the lateral (29%) and third ventricles (22%) [21]. In this case, a 1.4 cm × 1.4 cm × 2.2 cm mass in the fourth ventricle and caudal part of the brainstem showed characteristic CPT imaging features, including T2 hyperintensity and T1 hypointensity, with an internal hematoma appearing isointense on both sequences. Based on the characteristic MRI findings and anatomical location, CPT was strongly considered the primary differential diagnosis, with meningioma and glioma considered less likely. However, histopathological examination confirmed the tumor as a glioma. This indicates the limitations of imaging-based diagnosis, as MRI demonstrates an approximately 70% accuracy in predicting primary brain tumor types [4]. Therefore, glioma could also be considered in the differential diagnosis of intraventricular tumors [22].

Differentiating glioma subtypes using H&E staining alone is often difficult. While astrocytic nuclei are typically more elongated and angular than oligodendrocytic nuclei, such features can be obscured in high-grade tumors due to extensive necrosis and reduced cellularity [5,6,23]. In this case, severe necrosis and hemorrhage limited the number of observable tumor cells, making subtype classification based on H&E alone inconclusive. IHC is therefore essential for glioma subtyping. Olig2, GFAP, and vimentin are frequently used for glioma subtyping [6,8,24]. Both Olig2 and GFAP can be expressed in astrocytomas and oligodendrogliomas, whereas vimentin is more strongly expressed in astrocytomas [6,8]. Due to occasional positivity in oligodendrogliomas, its diagnostic specificity is limited [24]. To improve accuracy, additional markers such as PDGFRα, IGFBP2, EGFR, and CNPase have been investigated [6,8,23]. However, their expression may vary with tumor grade and fixation conditions, necessitating careful evaluation for accurate diagnosis [23]. Glioma grading has shifted from the WHO Grade I–IV system to a binary classification of low-grade and high-grade, based on key histologic features such as necrosis, microvascular proliferation, mitotic activity, and pseudopalisading [5,6,8,23].

Prognostic factors for glioma in humans include tumor grade, age, progression, extent of resection, and the use of adjuvant therapy [25,26]. The median survival time (MST) after glioma resection is 45.3–87 weeks in humans and approximately 24.7 weeks in dogs [27,28]. Given the prognostic factors, surgical removal may have extended the patient’s survival; however, the patient survived only 91 days postoperatively, indicating a significantly shorter survival duration. This may be attributed to incomplete resection and absence of adjuvant therapy. Previous studies have shown that reoperation and chemotherapy significantly improve survival in glioma patients, with one study reporting a median time to progression of 156 days and further benefit in reoperated patients [29,30]. Radiation therapy alone yields an MST of 9.4 months in humans, which increases to 12.0 months with combined chemotherapy [31]. Similarly, veterinary studies have shown that chemoradiation with agents like temozolomide or lomustine results in MSTs of 12–16.8 months, exceeding that of radiation alone (7.5–13 months) [5,30,32,33]. Thus, adjuvant chemotherapy or reoperation may have contributed to a more favorable outcome in this patient.

Extensive vascularization around the tumor led to significant intraoperative hemorrhage, requiring blood transfusion on estimated blood loss due to limited venous access from surgical positioning. Hemostasis was achieved using absorbable oxidized regenerated cellulose throughout the procedure and flowable thrombin material for final bleeding control. A previous study reported that this material effectively controlled intracranial hemorrhage even in patients with coagulation disorders [34]. Similarly, in this case, it provided immediate and effective hemostasis. Given its high cost and limited volume, flowable thrombin was reserved for final control. Despite transfusion targeting 24% PCV compensation, postoperative PCV decline suggested dilution and ongoing intraoperative loss. Therefore, significant hemorrhage could be considered during high-grade glioma resection, necessitating thorough preparation for transfusion.

Although the patient died 91 days postoperatively, the primary symptoms of ataxia and seizures were rapidly alleviated following surgery. This suggests that the telovelar approach may have significant clinical benefits in relieving neurological symptoms and improving quality of life in the case of caudal fossa gliomas. However, the invasive nature of malignant gliomas and the risk of significant intraoperative hemorrhage complicate surgical management. Therefore, this approach could be considered cautiously, with thorough preoperative planning and careful intraoperative management.

There are a few limitations in this case report. First, complete resection was limited due to the tumor’s deep location in the fourth ventricle, intraoperative bleeding, and its infiltrative nature, which made it difficult to distinguish from normal brain tissue. Second, analgesic options were limited due to strict regulations on opioids such as fentanyl and methadone in Korea. As butorphanol alone was considered insufficient, DLK CRI was selected as an alternative and proved effective for pain control. This suggested that DLK could be a viable option for peri- or postoperative pain control following brain surgery. Third, in this case, follow-up imaging was not performed after RT due to loss of contact with the owner, limiting assessment of treatment response and recurrence. If additional imaging had been performed, it could have allowed evaluation of treatment response, recurrence, and further treatment planning. Lastly, a lack of follow-up communication with the owner limited the assessment of the patient’s postoperative condition and long-term outcome. Postoperative mortality has been reported in approximately 13.1% of cases, particularly in patients with brainstem tumors requiring a suboccipital approach, with seizures contributing to delayed recovery and secondary brain injury, potentially leading to coma or death [35]. Neurological deterioration occurs in up to 60% of glioma and meningioma patients following radiation therapy, and 10% may die due to cardiovascular causes [36]. Further large-scale studies comparing brain tissue histopathology, MRI findings, and clinical outcomes, using resources such as brain tumor banks, are needed.

## 4. Conclusions

In this case, the telovelar approach was utilized for the resection of a glioma located in the fourth ventricle and the caudal part of the brainstem, presenting a viable surgical option for managing caudal fossa glioma. While complete resection was not possible because of the invasive nature of the brain tumor, significant debulking was successfully achieved. Debulking of the glioma relieved the pressure on the surrounding areas, leading to a noticeable improvement in the patient’s condition and good initial surgical results. However, further research is necessary to improve outcomes in similar cases.

## Data Availability

The original contributions of this study are presented in this article. Further inquiries can be directed to the corresponding authors.

## References

[1] José-López R., Gutierrez-Quintana R., de la Fuente C., Manzanilla E.G., Suñol A., Pi Castro D., Añor S., Sánchez-Masian D., Fernández-Flores F., Ricci E. (2021). Clinical features, diagnosis, and survival analysis of dogs with glioma. J. Vet. Intern. Med..

[2] Stoica G., Kim H.T., Hall D.G., Coates J.R. (2004). Morphology, immunohistochemistry, and genetic alterations in dog astrocytomas. Vet. Pathol..

[3] Snyder J.M., Shofer F.S., Van Winkle T.J., Massicotte C. (2006). Canine intracranial primary neoplasia: 173 cases (1986–2003). J. Vet. Intern. Med..

[4] Gladson C.L., Prayson R.A., Liu W.M. (2010). The pathobiology of glioma tumors. Annu. Rev. Pathol..

[5] Miller A.D., Miller C.R., Rossmeisl J.H. (2019). Canine primary intracranial cancer: A clinicopathologic and comparative review of glioma, meningioma, and choroid plexus tumors. Front. Oncol..

[6] Miller A.D., Porter B.F., Zachary J.F. (2022). Nervous System. Pathologic Basis of Veterinary Disease.

[7] Posti J.P., Bori M., Kauko T., Sankinen M., Nordberg J., Rahi M., Frantzén J., Vuorinen V., Sipilä J.O. (2015). Presenting symptoms of glioma in adults. Acta Neurol. Scand..

[8] Higgins R.J., Bollen A.W., Dickinson P.J., Sisó-Llonch S., Meunten D.J. (2017). Tumor of the Nervous System. Tumor in Domestic Animals.

[9] Antonakakis M.G., Carletti B.E., Anselmi C., McGrath S., Minguez J.J. (2022). Use of a telovelar approach for complete resection of a choroid plexus tumor in a dog. Vet. Surg..

[10] Deshmukh V.R., Figueiredo E.G., Deshmukh P., Crawford N.R., Preul M.C., Spetzler R.F. (2006). Quantification and comparison of telovelar and transvermian approaches to the fourth ventricle. Neurosurgery.

[11] Ebrahim K.S., Toubar A.F. (2019). Telovelar approach versus transvermian approach in management of fourth ventricular tumors. Egypt. J. Neurosurg..

[12] Yaşargil M.G., Abdulrauf S.I. (2008). Surgery of intraventricular tumors. Neurosurgery.

[13] El-Bahy K. (2005). Telovelar approach to the fourth ventricle: Operative findings and results in 16 cases. Acta Neurochir..

[14] Ghali M.G. (2021). Telovelar surgical approach. Neurosurg. Rev..

[15] Tomasello F., Conti A., Cardali S., La Torre D., Angileri F.F. (2015). Telovelar approach to fourth ventricle tumors: Highlights and limitations. World Neurosurg..

[16] Sufianov R., Pitskhelauri D., Bykanov A. (2022). Fourth ventricle tumors: A review of series treated with microsurgical technique. Front. Surg..

[17] Ezer H., Banerjee A.D., Bollam P., Guthikonda B., Nanda A. (2012). The superior transvelar approach to the fourth ventricle and brainstem. J. Neurol. Surg. Part B Skull Base.

[18] Jamshidi A.O., Priddy B., Beer-Furlan A., Prevedello D.M. (2019). Infradentate approach to the fourth ventricle. Oper. Neurosurg..

[19] Jeong J., Lee H., Rho Y., Jeon Y. (2024). Case report: Gross total resection of a primary fourth ventricular meningioma using the telovelar approach in a dog. Front. Vet. Sci..

[20] Cushing H. (1901). Concerning a definite regulatory mechanism of the vasomotor center which controls blood pressure during cerebral compression. Johnes Hopkins Hosp. Bull..

[21] Westworth D.R., Dickinson P.J., Vernau W., Johnson E.G., Bollen A.W., Kass P.H., Sturges B.K., Vernau K.M., Lecouteur R.A., Higgins R.J. (2008). Choroid plexus tumors in 56 dogs (1985–2007). J. Vet. Intern. Med..

[22] Omura T., Nawashiro H., Osada H., Shima K., Tsuda H., Shinsuke A. (2008). Pilomyxoid astrocytoma of the fourth ventricle in an adult. Acta Neurochir..

[23] Koehler J.W., Miller A.D., Miller C.R., Porter B., Aldape K., Beck J., Brat D., Cornax I., Corps K., Frank C. (2018). A revised diagnostic classification of canine glioma: Towards validation of the canine glioma patient as a naturally occurring preclinical model for human glioma. J. Neuropathol. Exp. Neurol..

[24] Fernández F., Deviers A., Dally C., Mogicato G., Delverdier M., Cauzinille L., Gnirs K., Añor S., de la Fuente C., Fondevila D. (2016). Presence of neural progenitors in spontaneous canine gliomas: A histopathological and immunohistochemical study of 20 cases. Vet. J..

[25] Pignatti F., Van Den Bent M., Curran D., Debruyne C., Sylvester R., Therasse P., Áfra D., Cornu P., Bolla M., Vecht C. (2002). Prognostic factors for survival in adult patients with cerebral low-grade glioma. J. Clin. Oncol..

[26] Wang J., Hu G., Quan X. (2019). Analysis of the factors affecting the prognosis of glioma patients. Open Med..

[27] Merickel J.L., Pluhar G.E., Rendahl A., O’Sullivan M.G. (2021). Prognostic histopathologic features of canine glial tumors. Vet. Pathol..

[28] Laws E.R., Parney I.F., Huang W., Anderson F., Morris A.M., Asher A., Lillehei K.O., Bernstein M., Brem H., Sloan A. (2003). Survival following surgery and prognostic factors for recently diagnosed malignant glioma: Data from the Glioma Outcomes Project. J. Neurosurg..

[29] Hidalgo Crespo E., Farré Mariné A., Pumarola I Battle M., Borrego Massó J.F., Luján Feliu-Pascual A. (2022). Survival time after surgical debulking and temozolomide adjuvant chemotherapy in canine intracranial gliomas. Vet. Sci..

[30] José-López R. (2023). Chemotherapy for the treatment of intracranial glioma in dogs. Front. Vet. Sci..

[31] Fine H.A., Dear K.B., Loeffler J.S., Black P.M., Canellos G.P. (1993). Meta-analysis of radiation therapy with and without adjuvant chemotherapy for malignant gliomas in adults. Cancer.

[32] Taal W., Bomberg J.E., van den Bent M.J. (2015). Chemotherapy in glioma. CNS Oncol..

[33] Trageser E., Martin T., Burdekin B., Hart C., Leary D., LaRue S., Boss M.K. (2023). Efficacy of stereotactic radiation therapy for the treatment of confirmed or presumed canine glioma. Vet. Comp. Oncol..

[34] Fiss I., Danne M., Stendel R. (2007). Use of gelatin-thrombin matrix hemostatic sealant in cranial neurosurgery. Neurol. Med. Chir..

[35] Kohler R.J., Arnold S.A., Eck D.J., Thomson C.B., Hunt M.A., Pluhar G.E. (2018). Incidence of and risk factors for major complications or death in dogs undergoing cytoreductive surgery for treatment of suspected primary intracranial masses. J. Am. Vet. Med. Assoc..

[36] Magalhaes T.R., Benoit J., NÉČovÁ S., Queiroga F.L. (2021). Outcome after radiation therapy in canine intracranial meningiomas or gliomas. In Vivo.

